# Dosimetric comparison of intensity-modulated proton therapy and proton arc therapy for pediatric ependymoma

**DOI:** 10.2340/1651-226X.2025.42001

**Published:** 2025-05-12

**Authors:** Helge Henjum, Karoline Mo Feten, Erlend Hartvigsen, Kristian S. Ytre-Hauge, Camilla G. Boer, Camilla H. Stokkevåg

**Affiliations:** a Department of Physics and Technology, University of Bergen, Bergen, Norway; b Department of Oncology and Medical Physics, Haukeland University Hospital, Bergen, Norway

**Keywords:** proton arc therapy, IMPT, robust optimization, robust evaluation, LET

## Abstract

**Background and purpose:**

Proton Arc Therapy (PAT) is an emerging proton therapy treatment modality with the potential to reduce radiation exposure to healthy tissues compared to conventional Intensity-Modulated Proton Therapy (IMPT) with fewer beams. This is an attractive option for treating pediatric patients, who are vulnerable to radiation-induced side effects. There is, however, a need to investigate the redistribution of dose to the target volume and organs at risk. In this study, we therefore explored the potential of PAT in proton therapy of pediatric ependymoma.

**Methods and materials:**

Three-field IMPT and PAT treatment plans for 10 pediatric ependymoma patients were optimized using the Eclipse treatment planning system. The PAT plans consisted of 8 fields, spanning 170 degrees. Both modalities were robustly optimized with a ± 2 mm isocenter shift and a ± 3% range uncertainty.

**Results:**

PAT showed improved CTV coverage compared to three-field IMPT, with a distinct increase in D_98%_. A clear dose reduction was found for the cochleae, with median values of 9.32 Gy(Relative Biological Effectiveness [RBE]) [0.76 – 30.40 Gy(RBE)] and 18.30 Gy(RBE) [1.24 – 29.75 Gy(RBE)] for PAT and IMPT, respectively, for the right cochlea. For the left cochlea, the respective doses were 12.34 Gy(RBE) [2.81 – 30.94 Gy(RBE)] and 18.49 Gy(RBE) [4.27 – 31.97 Gy(RBE)]. No significant difference for the brain integral dose was found between the two modalities.

**Interpretation:**

PAT can improve the dosimetric outcome of proton therapy in pediatric ependymoma patients. Organs at risk dose varied on a patient-to-patient basis; thus, individual treatment plan comparisons are recommended.

## Introduction

Proton Arc Therapy (PAT) is an emerging treatment delivery technology in proton therapy. PAT can deliver the dose from all angles by rotating the gantry around the patient, unlike conventional Intensity-Modulated Proton Therapy (IMPT), which uses only a few static fields [1].

When treating intracranial tumors, dosimetric conformity is crucial to provide sufficient dose to the tumor while sparing nearby critical organs at risks (OARs), such as the brainstem and optical structures. This is especially important when treating pediatric patients, as they are more susceptible to radiation-induced side effects [2, 3] compared to adults. Furthermore, studies on pediatric dose limits done by the PENTEC group showed an increased risk of cognitive disabilities with certain volume-dose relations, highlighting that certain dose limits should be pediatric specific. There is only a single comparative study between PAT and IMPT for pediatric patients, by Toussaint et al. [4], which found lower integral doses to a cohort of pediatric brain tumor patients from PAT, which led to a lower estimated risk of secondary primary cancers.

For whole brain radiotherapy in treatment of brain tumors, Ding et al. [5] found that PAT decreased the dose to the OARs, while maintaining target coverage. Conversely, in a study of an intracranial tumor by Sanchez-Parceriza [6], the integral dose for PAT was higher, compared to IMPT; however, these plans utilized mono- and bi-energetic proton beams. Furthermore, studies comparing PAT and IMPT have been conducted in lung cancer patients, where the integral dose and OAR doses were found to be lower for PAT, while maintaining or improving the target coverage [1, 6–9]. Similarly, head and neck tumors exhibited the same result, with a reduction in integral dose up to 21% [1, 10, 11]. Furthermore, Johnson et al. [12] further found no increase in secondary cancer risk for five different anatomical sites when comparing PAT and IMPT, including base of skull and head and neck cancers, further motivating the use of PAT.

There is a need for dosimetric studies to determine how PAT could enhance treatment outcomes for pediatric patients with intracranial tumors. In this study, we compared the dosimetric differences between IMPT and PAT in 10 pediatric ependymoma patients.

## Method

The Eclipse™ (Varian Medical System, Palo Alto, CA, USA) Treatment Planning System (TPS) was used to create three-field IMPT and PAT plans for 10 pediatric ependymoma patients. The number of patients selected is based on the availability of pediatric ependymoma patients with similar setups and is considered suitable for identifying differences across the studied treatment techniques and establishing a foundation for further research. The machine setting used for the planning was obtained from the Varian ProBeam 360 with a minimum monitor unit (MU) per spot of 0.012. All patient materials were anonymized, and the study materials were approved for use by the regional ethics committee. The prescribed dose for all patients was 54 Gy(Relative Biological Effectiveness [RBE]), given in 30 fractions, with a RBE of 1.1. The clinical goal was to achieve 95% of the prescription dose to 98% of the Clinical Target Volume (CTV) (D_98%_), and the OAR constraints applied were according to the European Particle Therapy Network (EPTN) [13] and the Danish neuro-oncology group [14]. All plans were normalized to achieve a median dose of 54 Gy(RBE) to the CTV. The doses were optimized using the nonlinear universal proton optimizer (NUPO) algorithm, and final doses were calculated with the proton convolution superposition (PCS) algorithm.

**Figure 1:**
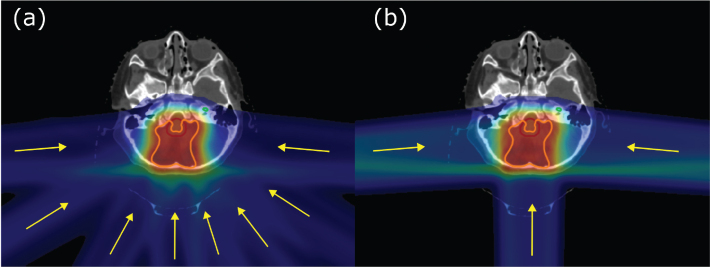
Example of field setup for (a) PAT and (b) IMPT, where the yellow lines represent the field setup. IMPT: Intensity-Modulated Proton Therapy; PAT: Proton Arc Therapy.

For the IMPT plans, two lateral opposing fields and an additional posterior field were utilized. The PAT plans utilized eight coplanar fields, forming a semi-arc of 170 degrees and avoiding irradiation of the anterior of the patient (Figure 1). The plans were robust optimized with a 3% range uncertainty and a 2 mm shift in isocenter, and in combination resulting in 20 total uncertainty scenarios. The robustness of the different plans was evaluated in terms of how many scenarios fulfilled our clinical goal of D_98%_ to the CTV.

Whereas linear energy transfer (LET) evaluation is emerging in proton therapy treatment protocols, the LET was calculated for the treatment plans using the FLUKA Monte Carlo code [14, 15]. Applying a user routine, the dose-averaged LET (LET_d_) to water from primary and secondary protons was calculated, as described by Grassberger and Paganetti [15]. LET_d_ values for all plans are reported in the Supplementary Materials.

The evaluation of the dosimetric differences between PAT and IMPT was done through CTV coverage, OAR-dose, integral dose, conformity index (CI), and homogeneity index (HI) (definitions found in the Supplementary Materials). The dose to OARs was evaluated based on the EPTN recommendation [13], the Danish neuro-oncology group [14], and also pediatric specific recommendations from the PENTEC group (Table A1). The integral dose to the brain was calculated as the product of the mean dose to the brain and the brain volume. IMPT and PAT were compared through the non-parametric Wilcoxon signed-rank test using the Python package scipy.stats.wilcoxon. Here, a *p*-value of less than 0.05 was considered statistically significant.

## Results

**Figure 2:**
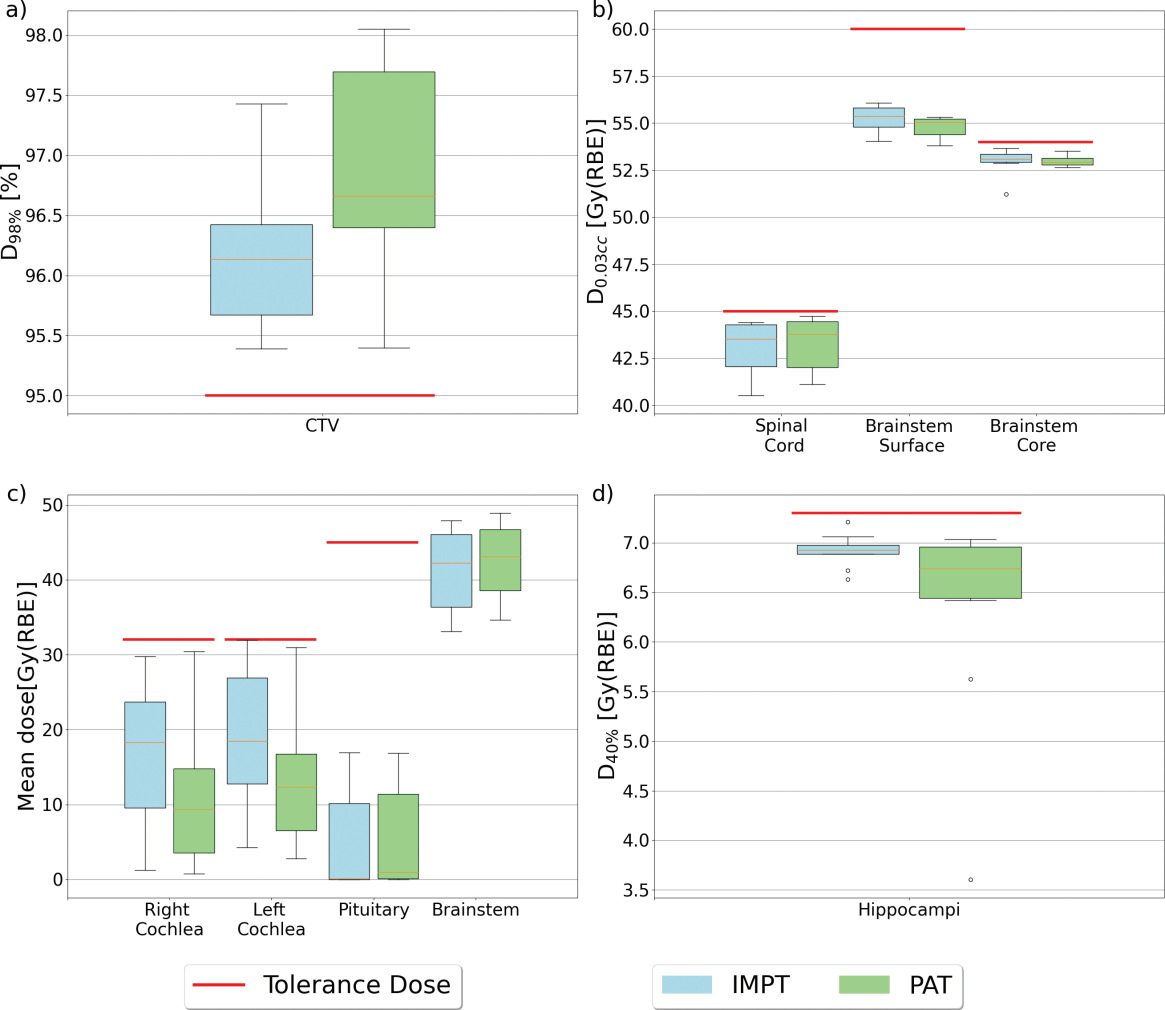
Boxplots for the different Region of Interest (ROIs). (a) The dose given to 98% of the CTV volume, (b) maximum doses to different OARs, (c) mean doses to different OARs, and (d) D_40%_ to the hippocampi. The red line represents the tolerance doses for the different ROIs. The top and bottom 25% of the observations are given over and under the box, the yellow line displays the median value, and the dots represent outliers. OAR: organs at risks.

The D_98%_ for the CTV was within the clinical goal of 95% of the prescribed dose for all patients, with 0.5 Gy(RBE) higher median dose for PAT than for IMPT (*p* < 0.01) (Figure 2). Furthermore, all PAT plans provided a higher D_98%_ for the CTV compared to their IMPT counterpart, except for one patient (Figure A1). The CI and HI were similar for both modalities, with the range of the HI being slightly higher for PAT (0.96 [0.94 – 0.97]) compared to IMPT (0.95 [0.93 – 0.96]) (Table A2 in the Supplementary Materials). The HI exhibited a statistically significant (*p*-value of 0.013) improvement for PAT (Figure A2).

**Figure 3:**
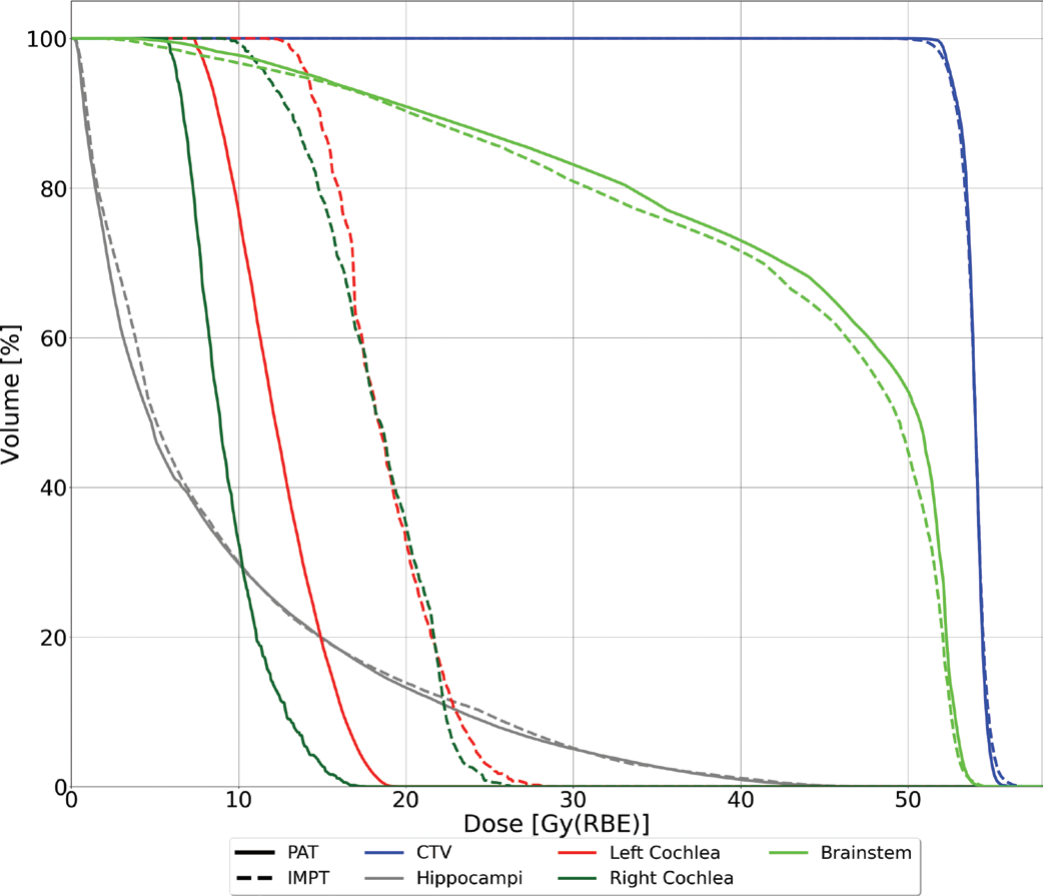
Median Dose Volume Histogram (DVH) for all patients. Solid lines represent PAT plans, while dashed lines represent IMPT plans. IMPT: Intensity-Modulated Proton Therapy; PAT: Proton Arc Therapy.

For the OARs, the dose difference varied across the structures (Figure 3). Both cochleae showed a significant reduction in mean dose with PAT compared to IMPT with median values of 9.32 Gy(RBE) [0.76 – 30.40 Gy(RBE)] and 18.30 Gy(RBE) [1.24 – 29.75 Gy(RBE)] for PAT and IMPT, respectively, for the right cochlea (*p* < 0.01), and 12.34 Gy(RBE) [2.81 – 30.94 Gy(RBE)] and 18.49 Gy(RBE) [4.27 – 31.97 Gy(RBE)] for the left cochlea (*p* < 0.01). Similarly, for the brainstem surface and core, a reduction for PAT was found, with a median max dose (d_0.03cc_) to the brainstem core of 55.04 Gy(RBE) [53.79 – 55.32 Gy(RBE)] and 55.37 Gy(RBE) [54.04 – 56.07 Gy(RBE)] for PAT and IMPT, respectively, and 52.89 Gy(RBE) [52.63 – 53.52] and 53.09 Gy(RBE) [51.22 – 53.66 Gy(RBE)] for the brainstem surface. Even though this reduction was smaller than the cochlea, it is evident from the range of doses (Figure 2) and the max doses from each patient (Figure A3 and Figure A4 in the Supplementary Materials) that overall doses were lower, especially for the brainstem surface. However, only the difference in surface dose was statistically significant (*p* < 0.01), and the brainstem received a statistically significant higher mean dose for the PAT plans compared to IMPT, with a median increase of 0.81 Gy(RBE) (*p* < 0.01).

For the spinal cord, PAT showed the highest dose, with PAT and IMPT providing a median d_0.03cc_ of 43.78 Gy(RBE)[41.11 – 44.73 Gy(RBE)] and 43.54 Gy(RBE) [40.52 – 44.41 Gy(RBE)], respectively (Figure 2). This is a relatively small increase and not statistically significant (*p* = 0.093). For the hippocampi (Figure 2d), the median D_40%_ was similar; however, the range was substantially lower for PAT [3.61 – 7.03 Gy(RBE)] compared to IMPT [6.63 – 7.21 Gy(RBE)].

**Figure 4:**
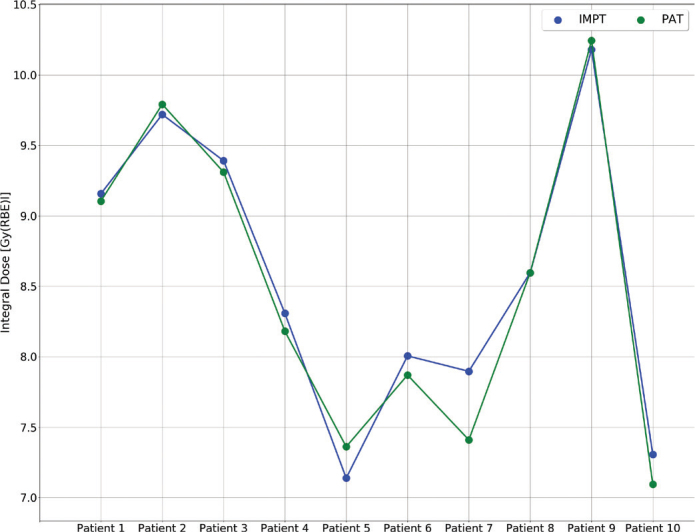
Integral dose to the brain for each patient for IMPT (green) and PAT (blue). IMPT: Intensity-Modulated Proton Therapy; PAT: Proton Arc Therapy.

The integral doses to the body structure and brain were similar for IMPT and PAT (*p* > 0.1), with prominent variation in integral dose from patient to patient (Figure 4). For the whole brain, the parameters d_10%_ and d_20%_ showed similar results for both modalities, while d50% and d100% were zero for both. However, when evaluating the maximum dose, PAT showed significantly lower doses, with 56.70 [56.11 – 57.51] and 57.40 [56.86 – 59.08] for PAT and IMPT, respectively (*p* < 0.01). Dose metrics for the CTV and OARs can be found in Table A2 in the Supplementary Materials.

Regarding the LET_d_ differences, PAT provided a slightly higher mean LET_d_ to the CTV, with a median value of 3.01 keV/µm [2.83 – 3.37 keV/µm] compared to IMPT (2.96 keV/µm ([2.69 – 3.21 keV/µm]) (*p* < 0.01). PAT also showed a reduction in LET_d_ for the brainstem, with median max LET_d_ of 7.71 keV/µm [6.70 – 9.34 keV/µm] and 8.92 [keV/µm] [6.71 – 11.67 keV/µm] (*p* < 0.01) for PAT and IMPT, respectively. All LET_d_ results can be found in Tables A3 and A4.

**Figure 5:**
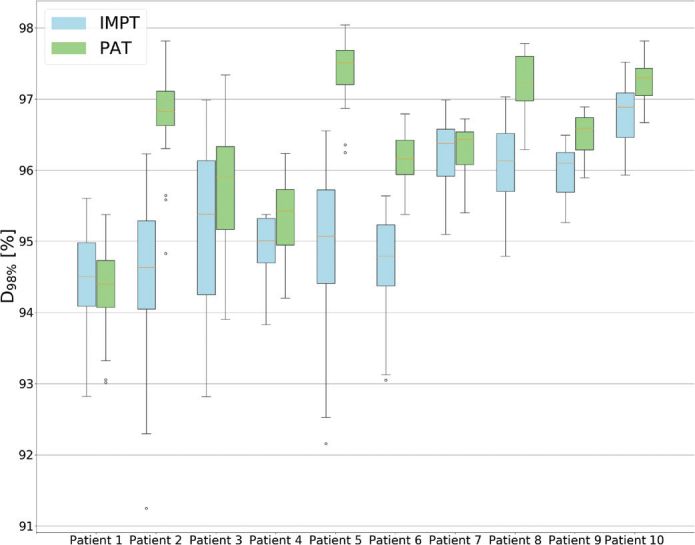
Boxplots showing the CTV coverage for each robust scenario for each patient. The top and bottom 25% of the observations are given over and under the box, the yellow line displays the median value, and the dots represent outliers.

In terms of robustness, the PAT plans provided an overall higher D_98%_ for the different robust scenarios compared to the IMPT plans (Figure 5). Furthermore, the total number of scenarios where the D_98%_ was above 95% (Table 1 and Figure A5) were 172 and 133 out of 200 for PAT and IMPT, respectively, representing a significant higher pass rate. The IMPT plans also provided the lowest D_98%_ for the worst-case scenarios, except for one patient (Table 1).

**Table 1:** Dose metrics and parameters for the different robust scenarios for each patient.

Patient	IMPT Scenarios where D_98%_ > 95% (out of 20)	PAT Scenarios where D_98%_ > 95% (out of 20)	IMPT Minimum D_98%_[%]	PAT Minimum D_98%_[%]	IMPT Minimum V_95%_[%]	PAT Minimum V_95%_[%]
**Patient 1**	5 (25%)	2 (00%)	92.82	**93.02**	96.15	**95.63**
**Patient 2**	8 (40%)	19 (95%)	**91.25**	94.83	94.18	97.86
**Patient 3**	13 (65%)	16 (80%)	92.82	93.90	95.69	96.29
**Patient 4**	10 (50%)	15 (75%)	93.83	94.20	96.43	97.38
**Patient 5**	11 (55%)	20 (100%)	92.16	96.25	**94.08**	98.87
**Patient 6**	7 (35%)	20 (100%)	93.05	95.38	95.09	98.27
**Patient 7**	20 (100%)	20 (100%)	95.10	95.40	98.14	98.57
**Patient 8**	19 (95%)	20 (100%)	94.79	96.29	97.88	98.68
**Patient 9**	20 (100%)	20 (100%)	95.26	95.90	98.46	99.19
**Patient 10**	20 (100%)	20 (100%)	95.93	96.67	98.93	99.49
**Sum**	133 of 200 (67%)	172 of 200 (86%)	N/A	N/A	N/A	N/A

The bold numbers represent the global minimum for all patients. IMPT: Intensity-Modulated Proton Therapy; PAT: Proton Arc Therapy.

## Discussion

In this study, IMPT and PAT plans were compared for 10 pediatric ependymoma patients. Clear differences in OAR doses were found as well as an improved tumor coverage and robustness for PAT plans.

A major motivation for PAT is the lower doses to OARs and the lower integral dose compared to IMPT with fewer beams. In this study, significantly lower doses were found for certain OARs by using PAT compared to IMPT. Nevertheless, IMPT achieved doses within the recommended tolerance limit [13] for all OARs, and the spinal cord received a slightly higher dose for the PAT plans. In comparison to other studies, Johnson et al. [12] compared PAT and IMPT for several tumor sites, including base of skull, where similar variations between the different OARs were found. This indicates that doses to OARs will vary between the different modalities and must be evaluated individually. They did, however, find that a transition from IMPT to PAT would not affect the secondary cancer (SC) risk. Furthermore, in the Toussaint *et al.*’s study [4], five pediatric brain tumor patients showed lower SC risk for PAT compared to IMPT.

We found a significant reduction in the mean dose to the cochlea with PAT compared to IMPT (median reduction of 11.4 and 16.65 Gy(RBE) for the left and right cochlea, respectively). Similarly, a reduction in dose in both cochleae and hippocampi was found in a study by Ding et al. [5], where PAT and IMPT were compared for whole brain irradiation. The EPTN [13] presented a consensus for dose constraints for OARs in neuro-oncology, where the mean dose for tinnitus and hearing loss was given at ≤32 Gy(RBE) and ≤45 Gy(RBE), respectively, and pediatric specific limits for risk of grade 3+ hearing loss given at ≤35 Gy(RBE) [16, 17]. However, as there is no clear dose threshold for hearing loss, the ‘as low as reasonably achievable’ (ALARA) principle applies [13]. Additionally, hearing loss was reported in up to 44% of patients receiving radiotherapy in cases of treatment beams passing through the inner ear [18]. This risk may be mitigated by PAT’s numerous beam angles, which disperses the dose and lowers the entrance dose for a single beam. Furthermore, in this study, the standard 3-field IMPT used co-lateral beams and a posterior field for treatment. The co-lateral fields will, in most cases, pass through the cochlea, which will increase the dose for the IMPT modality. Furthermore, these beam-angle effects are somewhat mitigated when utilizing PAT, where the entrance dose is increasingly spread around the target, lowering the dose levels for most OARs.

The PAT plans in this study are so-called static arcs, where the beam is not continuously rotated but delivering fields at different fixed angles. This would, in most cases, require longer delivery time, as more energy layer switching is required. It has, however, been shown that energy layer reduction algorithm can be applied to reduce delivery time with static arc delivery [10]. Here, it was shown that by selecting initial and final number of energy layers, delivery time could be reduced significantly while maintaining a robust plan. Whereas beam delivery techniques and times are rapidly evolving and a focus of many studies [10, 19–21], the primary goal of our study was to investigate the clinical potential with respect to adding more fields, i.e. 8-field IMPT and 3-field IMPT.

No difference in integral dose between PAT and IMPT was found in this study. This is in contrast to recent studies on head and neck tumors [10] and brain tumors [4], which found a reduction in integral dose of 21% and 17%, respectively. Both studies did, however, dispose an entire 360-degree arc in their planning in contrast to the semi arcs used in this study, which may impact the integral dose. The comprehensive review from the PENTEC group recently revealed certain pediatric specific dose constraints to the brain, where cognitive decline is the endpoint. Potential risk for cognitive brain damage is measured according to PENTEC, which is max dose of 59 Gy(RBE), while a variable dose-volume relationship also contributes to this. This dose-volume relationship limits 10% of the brain receiving over 36 Gy(RBE), 20% of the brain receiving over 29 Gy(RBE), 50% of the brain receiving over 22 Gy(RBE), and 100% of the brain receiving over 18 Gy(RBE) [22]. Only a single patient received over the maximum dose threshold, while PAT showed lower maximum doses, which might indicate a lower risk of cognitive-related side effects occurring with PAT (*p* < 0.01). Furthermore, using PAT, the doses are more spread out, meaning hot spots of dose due to high LET, in general, may be diminished, as seen in the Toussaint et al.’s study [23], where increasing number of beams resulted in lower maximum LET values. This is further illustrated in this study, where a reduction in high doses and LET values for e.g. the brainstem was observed.

The results indicated that the tumor coverage can be improved with PAT, as the D_98%_ was improved for 9/10 patients. In particular, a higher range of HI values were found among PAT plans. A recent review by Carabe-Fernandez [24] showed no difference in HI and CI across PAT and IMPT for brain tumor cases, prostate cases, and Head and Neck cases, while a slight improvement with PAT was found for lung cancers. However, in the reviewed brain tumor and lung case by Sanchez-Parcerisa [6], mono and bi-energetic beams were used, which could have affected the outcome. In recent years, several studies have found significant differences in CI and HI. Cao et al. [25] used Intensity-Modulated PAT to achieve a significant improved HI and CI for four ependymoma patients compared to IMPT, and similarly, de Jong et al. [10] found superior HI and CI for PAT plans in oropharyngeal cancer patients. This shows that PAT can also achieve superior tumor coverage, which is a parameter often overlooked, as the main rationale for PAT is the OAR sparing.

In this study, we assumed a clinical RBE of 1.1; however, it is widely known to vary depending on several factors, such as tissue type and the LET. Now, there is no consensus on how to implement this variable RBE clinically, although most models show that a higher LET will correspond to a higher RBE. Several studies have compared the LET between IMPT and PAT where PAT shows a general higher LET in the target volume, illustrating a higher biological effect [26–29]. Even though there is no consensus on how to evaluate LET clinically, a higher LET in target compared to IMPT was also found for PAT in this study, which further shows the potential for PAT in terms of biological dose. An increase in maximum LET in the brainstem was also found for IMPT compared to PAT, which could indicate a higher potential risk of necrosis, as a correlation between LET and brain toxicity has been indicated in several studies [13–16].

Robust optimization is slowly becoming the gold standard in treatment planning of protons and is considered a safer option compared to its planning target volume (PTV)-counterpart. The PAT plans provided more robust plans compared to IMPT, both in terms of robust scenarios passing the clinical goals and concerning the worst-case scenario for D_98%_ in the CTV. Liu et al. [30] evaluated the robustness, e.g. through root mean square deviation, which has been further used as an evaluation criteria for studies including the SPArc algorithm [5, 8, 9, 31, 32]. In these studies, similar or improved robustness has been found using SPArc compared to IMPT, highlighting improved robustness from PAT in general. Furthermore, a recent study by Tattenberg et al. [33] demonstrated an improved Normal Tissue Complication Probability (NTCP) from PAT compared to IMPT when applying two sets of range uncertainties. However, in a study by Argota-Perez et al. [34], even though a dosimetric improvement in head and neck tumors was found for PAT, these plans were less robust toward daily anatomical changes.

## Conclusion

The comparison across PAT and three-field IMPT demonstrated that PAT generally delivered lower OAR doses, provided improved robustness and target coverage. The OAR doses still varied across the patients; thus, individual treatment plan comparisons are recommended. Both modalities provided similar integral doses to the brain, and a slight improvement in LETd for PAT was observed.

## Supplementary Material



## Data Availability

The raw data supporting the conclusions of this article will be made available by the authors, without undue reservation.
