# Supplementary material for: Dosimetric comparison of intensity-modulated proton therapy and proton arc therapy for pediatric ependymoma
Source: Acta Oncol. 2025 May 12;64:42001. doi: 10.2340/1651-226X.2025.42001 (PMC12086448; doi:10.2340/1651-226X.2025.42001)
Supplement: Supplementary file 1 [file AO-64-42001-s1.pdf]

Supplementary material has been published as submitted. It has not been copyedited, or typeset by Acta Oncologica

**Supplementary Materials**

In Table A1, the constraints for the different OARs used in this study are found.

*Table A1 Overview of OAR dose constraints used in the planning process. All values are from EPTN recommendations, except for the spinal cord, which are from DNOG*

| Organ | Dose Constraint |
|---|---|
| **Brain** | $V_{60Gy} < 3cc$ |
| **Brainstem** | $\text{Surface}: D_{0.03cc} \leq 60\text{ Gy}$ |
| **Brainstem** | $\text{Core}: D_{0.03cc} \leq 54\text{ Gy}$ |
| **Chiasm** | $D_{0.03cc} \leq 55\text{ Gy}$ |
| **Cochlea** | $D_{mean} \leq 32\text{ Gy}$ |
| **Hippocampi** | $D_{40\%} \leq 7.3\text{ Gy}$ |
| **Pituitary** | $D_{mean} \leq 45\text{ Gy}$ |
| **Pituitary** | $D_{mean} \leq 20\text{ Gy}$ |
| **Spinal Cord** | $D_{0.03cc} \leq 45\text{ Gy}$ |

There are several definitions for conformity index (CI) in the literature (Feuvret et al. 2006). In this study we have used the definition by van’t Riet et al (van't Riet et al. 1997) , but we include other models by RTOG (Shaw et al. 2000), the SALT group (Dejean et al. 2001) and Lomax *et al* (Lomax and Scheib 2003), for comparison. Below are the definitions of CI and HI used in this study, and corresponding values can be found in Table A2:

$$CI_{van't\ Riet} = \frac{V_{95\%,CTV} * V_{95\%,CTV}}{V_{95\%,BODY} * V_{CTV}}$$

$$CI_{RTOG} = \frac{V_{95\%,BODY}}{V_{CTV}}$$

$$CI_{SALT} = \frac{V_{95\%,CTV}}{V_{CTV}}$$

$$CI_{Lomax} = \frac{V_{95\%,CTV}}{V_{95\%,BODY}}$$

$$Homogeneity\ Index = \frac{D_{5\%,CTV}}{D_{95\%,CTV}}$$

Table A2 show all dose metrics for the different regions of interest, while Table A3 shows the different median LET values found for the different structures for all patients. A dose cutoff of 2Gy (RBE) was used.

*Table A2 Dose metrics for IMPT and PAT. The median of the metrics with the corresponding range are given, and the statistical significance of the results are given with the p-value.*

| Structure | Metric | IMPT [Median(range)] | PAT [Median(range)] | p-value |
|---|---|---|---|---|
| **CTV** | $d_{98\%}$[%] | 96.13 (95.39 - 97.43) | 96.66 (95.40 - 98.05) | <0.01 |
| **CTV** | CI (RTOG) | 1.35(1.13 - 1.61) | 1.41(1.13 - 1.57) | >0.1 |
| **CTV** | CI (SALT) | 0.99(0.99 - 1.00) | 1.00(0.99 - 1.00) | <0.01 |
| **CTV** | CI (Lomax) | 0.73(0.62 - 0.88) | 0.71(0.64 - 0.88) | >0.1 |
| **CTV** | CI (van't Riet) | 0.73(0.62 - 0.87) | 0.71(0.64 - 0.88) | >0.1 |
| **CTV** | HI | 0.95 (0.93 - 0.96) | 0.96 (0.94 - 0.97) | 0.013 |
| **Hippocampi** | $d_{40\%}$[Gy(RBE)] | 6.93 (6.63 - 7.21) | 6.74 (3.61 - 7.03) | 0.060 |
| **Right Cochlea** | Mean[Gy(RBE)] | 18.30 (1.24 - 29.75) | 9.32 (0.76 - 30.40) | <0.01 |
| **Left Cochlea** | Mean[Gy(RBE)] | 18.49 (4.27 - 31.97) | 12.34 (2.81 - 30.94) | <0.01 |
| **Spinal cord** | $d_{0.03cc}$[Gy(RBE)] | 43.54 (40.52 - 44.41) | 43.78 (41.11 - 44.73) | 0.093 |
| **Pituitary** | Mean[Gy(RBE)] | 0.08 (0.00 - 16.90) | 0.97 (0.00 - 16.85) | 0.011 |
| **Brainstem** | Mean[Gy(RBE)] | 42.25 (33.10 - 47.90) | 43.06 (34.61 - 48.92) | <0.01 |
| **Brainstem Surface** | $d_{0.03cc}$[Gy(RBE)] | 55.37 (54.04 - 56.07) | 55.04 (53.79 - 55.32) | <0.01 |
| **Brainstem Core** | $d_{0.03cc}$[Gy(RBE)] | 53.09 (51.22 - 53.66) | 52.89 (52.63 - 53.52) | >0.1 |
| **Body** | ID [Gy(RBE)l] | 14.46 (9.75 - 19.99) | 14.49 (9.28 - 19.94) | >0.1 |
| **Brain** | ID [Gy(RBE)l] | 8.45 (7.14 - 10.18) | 8.39 (7.09 - 10.25) | >0.1 |

*Table A3 Median $LET_d$ values for all patients. Dose threshold were set for 2 Gy(RBE).*

| | $LET_d$ for IMPT $[keV/\mu m]$ | | | $LET_d$ for PAT $[keV/\mu m]$ | | |
|---|---|---|---|---|---|---|
| **Structure** | **Mean (range)** | **Min (range)** | **Max (range)** | **Mean (range)** | **Min (range)** | **Max (range)** |
| **CTV** | 2.96 (2.69 - 3.21) | 2.25 (1.54 - 2.64) | 5.96 (4.74 - 9.85) | 3.01 (2.83 - 3.37) | 2.21 (1.53 - 2.74) | 5.90 (4.99 - 9.14) |
| **Hippocampi** | 2.70 (1.28 - 3.40) | 0.00 (0.00 - 0.00) | 7.50 (4.51 - 9.02) | 2.48 (1.15 - 2.74) | 0.00 (0.00 - 0.00) | 7.08 (4.57 - 8.30) |
| **Right Cochlea** | 3.99 (0.33 - 5.61) | 3.01 (0.00 - 4.86) | 4.39 (1.32 - 7.32) | 4.14 (0.02 - 5.62) | 2.70 (0.00 - 5.38) | 4.65 (0.32 - 5.79) |
| **Left Cochlea** | 4.58 (1.19 - 5.24) | 3.16 (1.12 - 4.73) | 5.43 (1.37 - 7.49) | 4.00 (1.24 - 5.75) | 3.61 (0.00 - 5.48) | 5.44 (1.47 - 7.15) |
| **Spinal Cord** | 1.48 (0.83 - 4.07) | 0.00 (0.00 - 3.20) | 5.57 (4.71 - 7.54) | 1.83 (0.94 - 3.58) | 0.00 (0.00 - 2.85) | 5.95 (4.46 - 8.90) |
| **Pituitary** | 0.00 (0.00 - 5.57) | 0.00 (0.00 - 4.42) | 0.00 (0.00 - 7.22) | 0.01 (0.00 - 6.31) | 0.00 (0.00 - 5.68) | 0.18 (0.00 - 8.04) |
| **Brainstem** | 3.73 (3.38 - 5.34) | 1.04 (0.00 - 2.61) | 8.92 (6.71 - 11.67) | 3.99 (3.52 - 4.72) | 0.94 (0.00 - 2.67) | 7.71 (6.70 - 9.34) |

*Table A4 Corresponding p-values for the differences in table A4*

| Structure | p-value for mean $LET_d$ | p-value for min $LET_d$ | p-value for max $LET_d$ |
|---|---|---|---|
| **CTV** | <0.01 | >0.1 | >0.1 |
| **Hippocampi** | >0.1 | >0.1 | >0.1 |
| **Right Cochlea** | >0.1 | >0.1 | >0.1 |
| **Left Cochlea** | >0.1 | >0.1 | >0.1 |
| **Spinal Cord** | >0.1 | >0.1 | >0.1 |
| **Pituitary** | 0.043 | >0.1 | 0.043 |
| **Brainstem** | >0.1 | >0.1 | <0.01 |

Figure A1 and Figure A2 shows the $D_{98\%}$ values and HI for each patient, respectively. Figure A3 and Figure A4 show the maximum doses to the brainstem surface and core, respectively, for each patient, while Figure A5 shows $D_{98\%}$ for all robust scenarios for each patient.

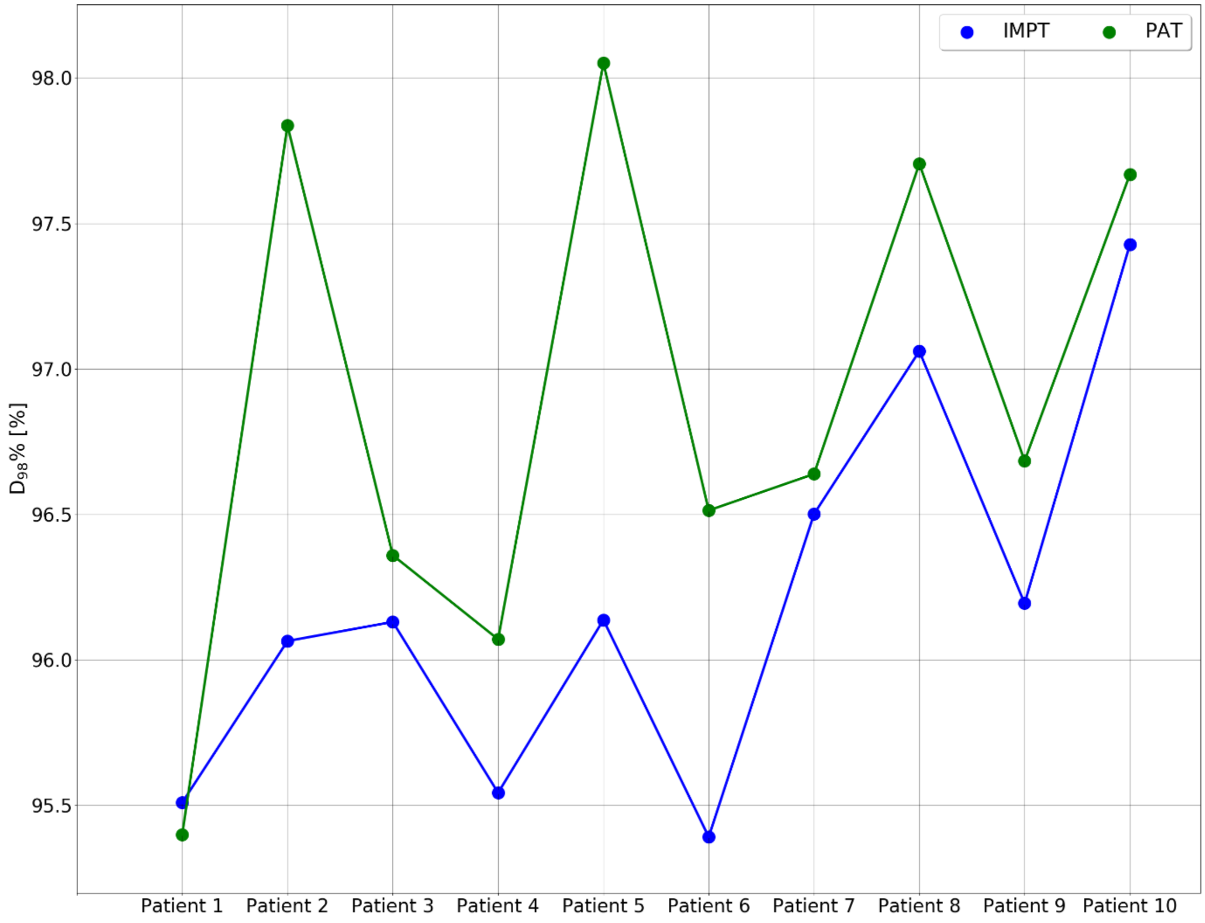


*Figure A1 The $D_{98\%}$ for each patients for both IMPT (blue) and PAT (green).*

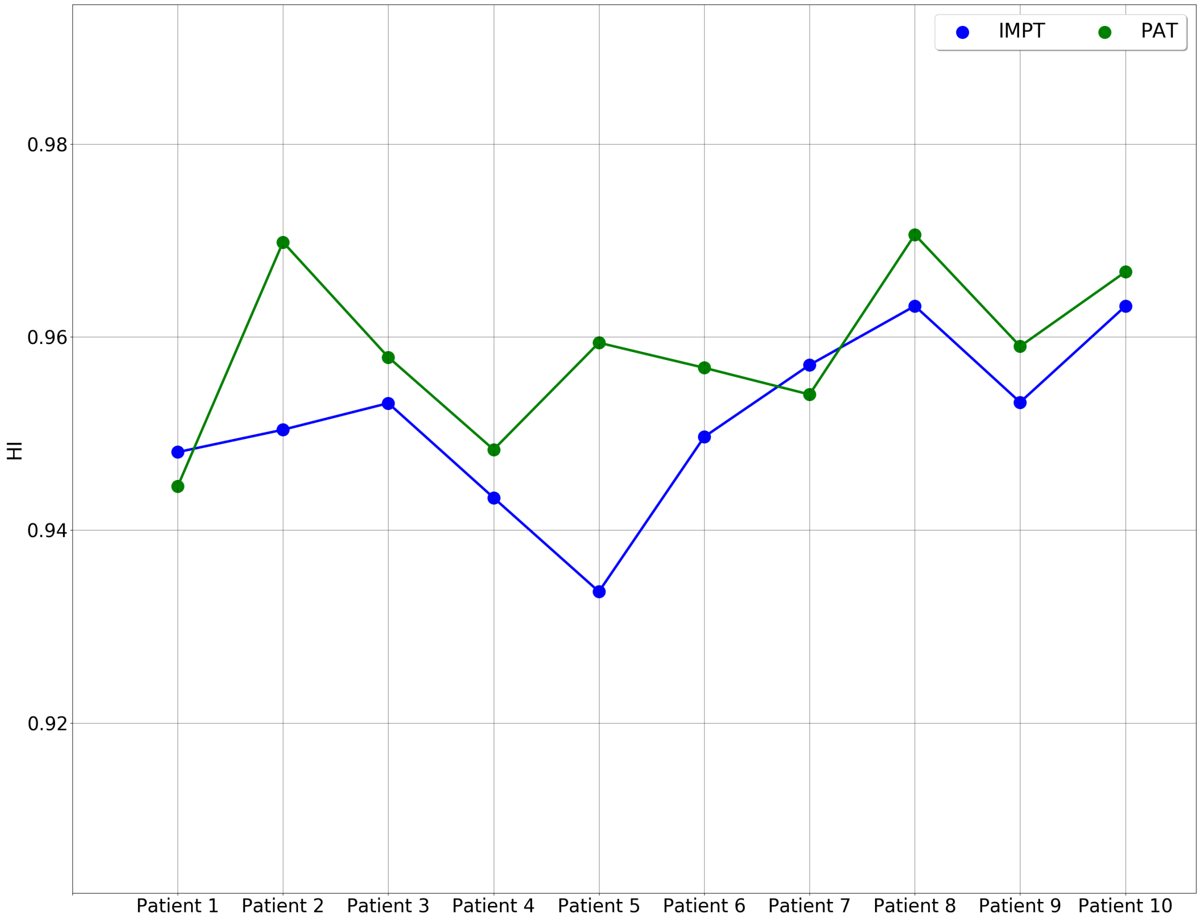


*Figure A2 HI for all patients for both IMPT (blue) and PAT (green).*

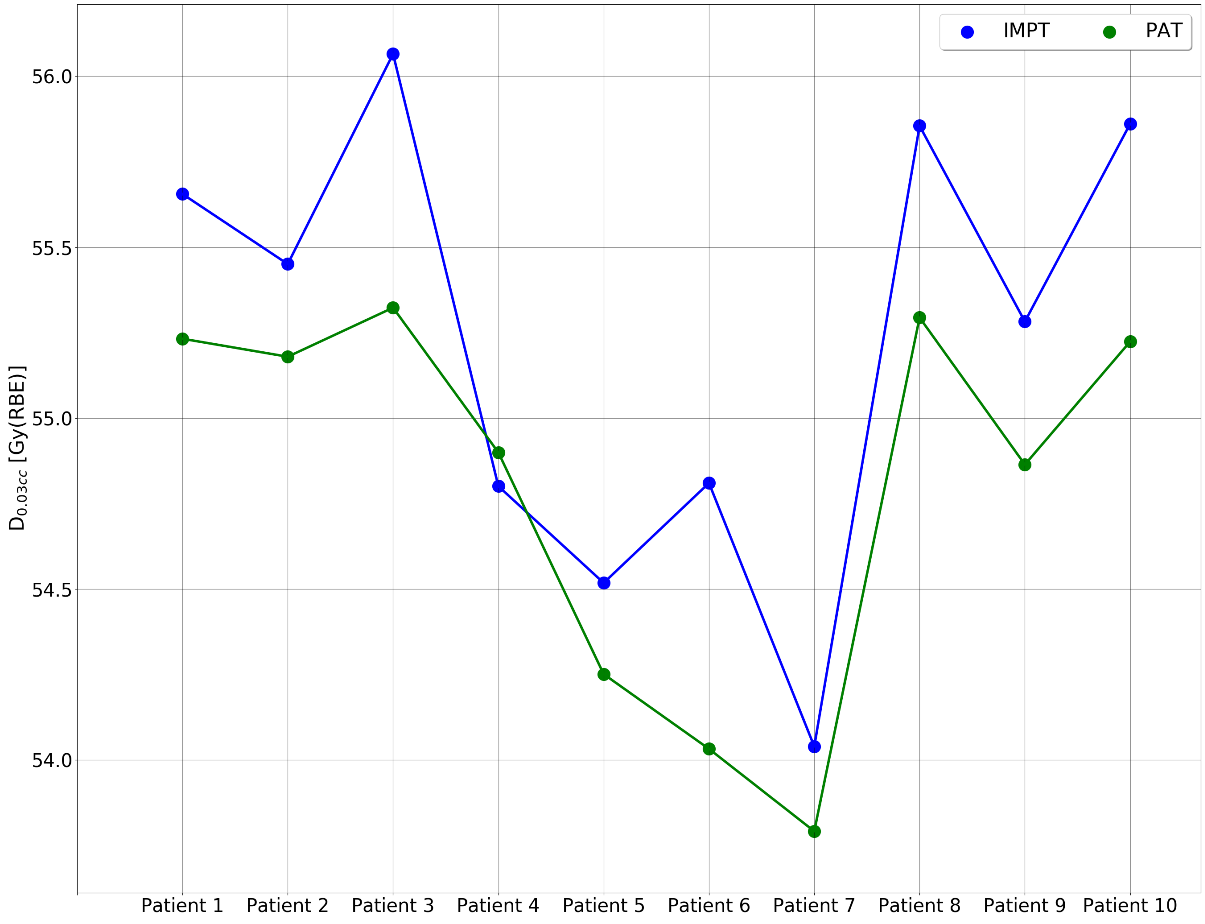


*Figure A3 The maximum dose to the brainstem core for each patient. The green dots represent the PAT plans, while the blue dots represent the IMPT plans.*

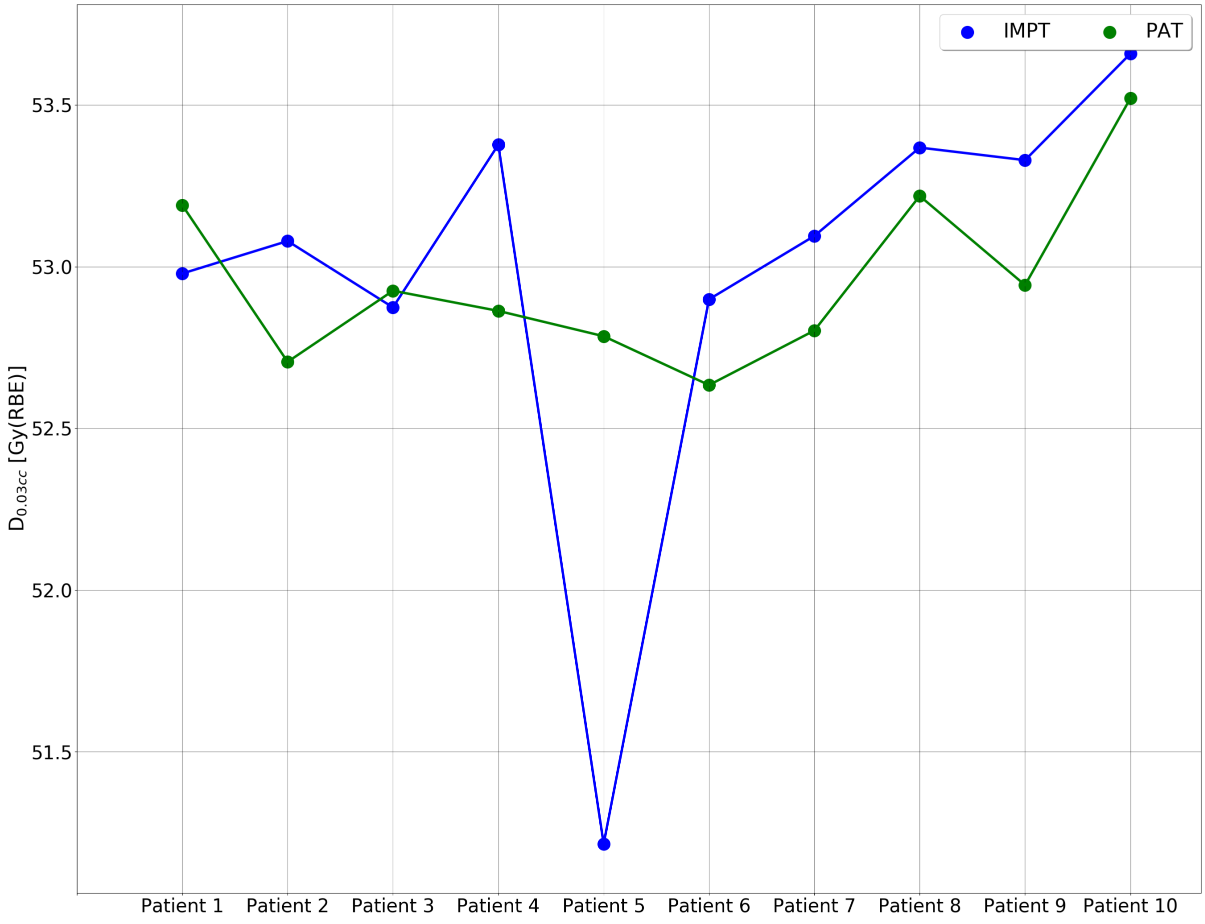


*Figure A4 The maximum dose to the brainstem core for each patient. The green dots represent the PAT plans, while the blue dots represent the IMPT plans.*

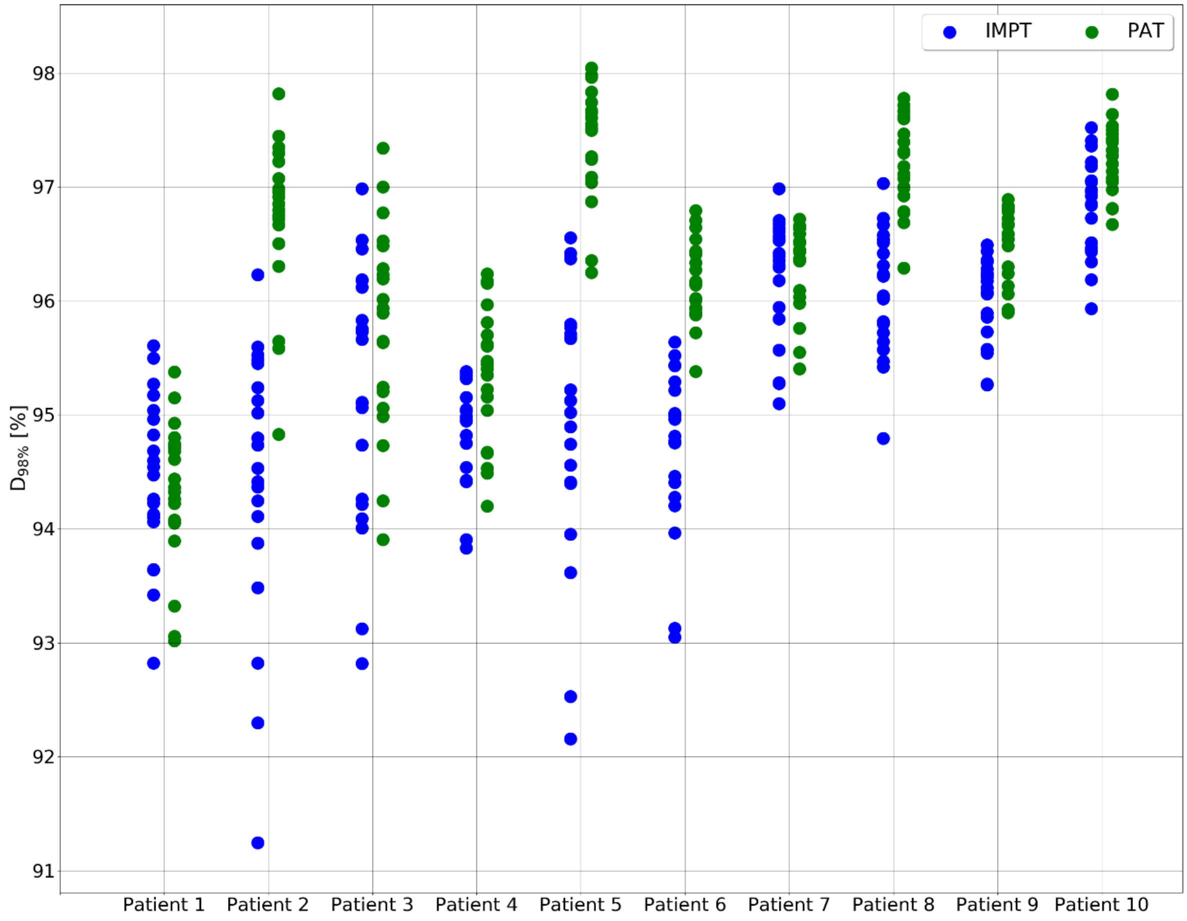


*Figure A5 The $D_{98\%}$ for all robust scenarios for each patient. The green dots represent the PAT plans, while the blue dots represent the IMPT plans.*

Dejean, C., D. Lefkopoulos, J. N. Foulquier, M. Schlienger, and E. Touboul. 2001. '[Automatic definition of prescription isodose for stereotaxic radiation of arteriovenous malformations]', *Cancer Radiother*, 5: 138-49.

Feuvret, L., G. Noel, J. J. Mazeron, and P. Bey. 2006. 'Conformity index: a review', *Int J Radiat Oncol Biol Phys*, 64: 333-42.

Lomax, N. J., and S. G. Scheib. 2003. 'Quantifying the degree of conformity in radiosurgery treatment planning', *Int J Radiat Oncol Biol Phys*, 55: 1409-19.

Shaw, E., C. Scott, L. Souhami, R. Dinapoli, R. Kline, J. Loeffler, and N. Farnan. 2000. 'Single dose radiosurgical treatment of recurrent previously irradiated primary brain tumors and brain metastases: final report of RTOG protocol 90-05', *Int J Radiat Oncol Biol Phys*, 47: 291-8.

van't Riet, A., A. C. Mak, M. A. Moerland, L. H. Elders, and W. van der Zee. 1997. 'A conformation number to quantify the degree of conformality in brachytherapy and external beam irradiation: application to the prostate', *Int J Radiat Oncol Biol Phys*, 37: 731-6.
